# Joint External Evaluation—Development and Scale-Up of Global Multisectoral Health Capacity Evaluation Process

**DOI:** 10.3201/eid2313.170949

**Published:** 2017-12

**Authors:** Elizabeth Bell, Jordan W. Tappero, Kashef Ijaz, Maureen Bartee, Jose Fernandez, Hannah Burris, Karen Sliter, Simo Nikkari, Stella Chungong, Guenael Rodier, Hamid Jafari, the CDC JEE Team and WHO Geneva JEE Secretariat

**Affiliations:** Centers for Disease Control and Prevention, Atlanta, Georgia, USA (E. Bell, J.W. Tappero, K. Ijaz, M. Bartee, H. Jafari);; US Department of Health and Human Services Office of Global Affairs, Washington, DC, USA (J. Fernandez, H. Burris);; US Department of Agriculture Animal and Plant Health Inspection Service, Washington (K. Sliter);; Center for Biothreat Preparedness, Helsinki, Finland (S. Nikkari);; World Health Organization Health Emergencies Programme, Geneva, Switzerland (S. Chunong, G. Rodier)

**Keywords:** Joint External Evaluation, Global Health Security Agenda, International Health Regulations, global health, global health capacity

## Abstract

The Joint External Evaluation (JEE), a consolidation of the World Health Organization (WHO) International Health Regulations 2005 (IHR 2005) Monitoring and Evaluation Framework and the Global Health Security Agenda country assessment tool, is an objective, voluntary, independent peer-to-peer multisectoral assessment of a country’s health security preparedness and response capacity across 19 IHR technical areas. WHO approved the standardized JEE tool in February 2016. The JEE process is wholly transparent; countries request a JEE and are encouraged to make its findings public. Donors (e.g., member states, public and private partners, and other public health institutions) can support countries in addressing identified JEE gaps, and implementing country-led national action plans for health security. Through July 2017, 52 JEEs were completed, and 25 more countries were scheduled across WHO’s 6 regions. JEEs facilitate progress toward IHR 2005 implementation, thereby building trust and mutual accountability among countries to detect and respond to public health threats.

In consideration of the growth in international travel and trade, the emergence and reemergence of international disease threats, and other public health risks, in 1995 the 48th World Health Assembly called for a substantial revision of the International Health Regulations (IHR). The 2003 severe acute respiratory syndrome (SARS) outbreak led to the rapid spread of the SARS coronavirus across 4 continents, resulting in 8,098 cases and 774 deaths (*1*). The failure to contain SARS at its source gave new momentum to amending the IHR, resulting in adoption of the revised IHR in May 2005 (IHR 2005) that went into effect in June 2007 with the stated goal that all member states self-report annually on their progress toward complying and that all member states would fully achieve compliance within 5 years (i.e., by mid-year 2012) (*2*,*3*). IHR 2005 is a legally binding instrument among all 196 World Health Organization (WHO) member states. Despite two 2-year extensions (2012 and 2014), by 2016, only one third of member states self-reported having attained IHR 2005 compliance (*4*)*.* In addition, although 195 of the states reported their compliance status at least once during the annual reporting period during 2010–2016, most member states failed to report annually on their progress toward compliance.

As a consequence, in November 2014, the IHR Review Committee on Second Extension for establishing national public health capacities and on IHR 2005 implementation recommended strengthening the self-assessment system, implementing in-depth reviews of events, and developing options “to move from self-evaluations to approaches that combine self-evaluation, peer review and voluntary external evaluation involving a combination of domestic and independent experts” (*5*). Following these recommendations, WHO developed an IHR 2005 monitoring and evaluation framework comprising 4 components: annual reporting, Joint External Evaluation (JEE), after-action review, and simulation exercise (*6*).

## Global Health Security Agenda (GHSA) and Independent External Country Assessments

The GHSA was launched in February 2014 at the US Department of Health and Human Services. It comprised representatives of 26 nations, WHO, the Food and Agriculture Organization of the United Nations (FAO), and the World Organisation for Animal Health (OIE) to prevent, detect, and respond to serious infectious disease threats with the capacity for rapid spread and to galvanize national efforts toward IHR 2005 compliance to prevent such diseases (*7*).

At the first GHSA Ministerial Meeting, hosted by the White House in September 2014, the GHSA Executive Steering Committee called for development of a comprehensive, independently administered monitoring and evaluation framework for GHSA. A GHSA evaluation tool would be used to establish a national baseline for GHSA capacities across the agenda’s 11 technical areas (also known as Action Packages) and to monitor progress of GHSA implementation over time. Six GHSA member nations (Republic of Georgia, Peru, Portugal, Uganda, United Kingdom, and Ukraine) volunteered to pilot the tool in their countries and to make the findings publicaly available on the GHSA website (https://www.ghsagenda.org/). The US Centers for Disease Control and Prevention (CDC) led the development of the GHSA monitoring and evaluation tool, working closely with Finland (the chair of the 2015 GHSA Executive Steering Committee), and a few subject matter experts (SMEs) from Georgia, Peru, Tanzania, Uganda, and the United Kingdom serving with >1 of GHSA’s 11 technical area working groups. The GHSA tool development also was informed by several existing monitoring and evaluation frameworks: the WHO IHR Annual Reporting Tool; OIE tool for the Evaluation of Performance of Veterinary Services; CDC’s Public Health Emergency Preparedness Performance Measures; Global Immunization Index; International Atomic Energy Agency Safety Assessment; and the WHO Ebola Virus Preparedness Checklist. WHO participated as an observer on several of the first 6 GHSA monitoring and evaluation assessments.

## WHO Joint External Evaluation Tool

In March 2015, as Ebola virus disease (EVD) threatened to spread from Guinea, Liberia, and Sierra Leone to other West Africa countries and beyond, WHO regional offices conducted Ebola assessment missions to independently assess the capacities of the countries to prevent, detect, and respond to a potential importation of EVD. The findings of the assessments highlighted gaps in the IHR 2005 core capacities for these countries in detecting, notifying, and responding to EVD that the annual self-reporting monitoring tool did not identify. Although the annual reporting tool serves a different purpose from the disease-specific checklist used during these EVD preparedness assessments, the results were a proxy measure of the capacity of the country to manage a specific outbreak. For example, in the Eastern Mediterranean Regional Office (EMRO), public health contingencies for points of entry were self-reported to be available in 84% of the countries assessed, but the Ebola assessment mission found that only 30% of countries had such contingency plans. Similarly, all countries assessed by data from the annual reporting self-reported the existence of IHR multisectoral committees, but the mission found multisectoral committees in only 25% of these countries. This finding provided evidence that the self-reporting of IHR capacities might not accurately reflect the actual capacities in some countries (*8**,**9*).

During 2015, external and independent GHSA assessments were completed in the 6 countries by rostered SMEs from WHO as observers and GHSA partnering countries; results were made publicaly available at the GHSA website. Lessons learned from these 6 GHSA pilot assessments informed revisions and the adoption of a final GHSA monitoring and evaluation tool, with results displayed in a tricolor (i.e., red, no capacity; yellow, limited capacity; green, full capacity) framework organized by technical area.

In January 2016, WHO convened a meeting with CDC and other GHSA partners in Cairo to integrate and standardize the existing IHR monitoring and evaluation tool with the GHSA external assessment tool. In February 2016, the WHO Secretariat and partners approved the consolidated voluntary JEE tool as part of the IHR Monitoring and Evaluation Framework (IHRMEF) across 19 core preparedness and response capacities for infectious disease, chemical, radiologic, and nuclear threats (*10*) (Table).

**Table:** JEE tool technical areas*

Element and technical areas	
Prevention	
1. National legislation, policy, and financing	
2. IHR 2005 coordination, communication, and advocacy	
3. Antimicrobial resistance in zoonotic disease	
4. Food safety	
5. Biosafety and biosecurity	
6. Immunization	
Detection	
8. National laboratory system	
9. Real-time surveillance	
10. Reporting	
11. Workforce development	
Response	
12. Preparedness	
13. Emergency Operations Centers	
14. Linking public health and security authorities	
15. Medical countermeasures and personnel deployment	
16. Risk communication	
Other hazards	
17. Points of entry	
18. Chemical events	
19. Radiation emergencies	

*The JEE tool incorporates all elements of the IHR 2005 (*2*) and the Global Health Security Agenda (https://www.ghsagenda.org/) assessment tool to evaluate a country’s capacity to prevent, detect, and respond to public health risks across 19 technical areas. JEE, Joint External Evaluation; IHR 2005, International Health Regulations 2005.

## JEE Process

**Figure 1:**
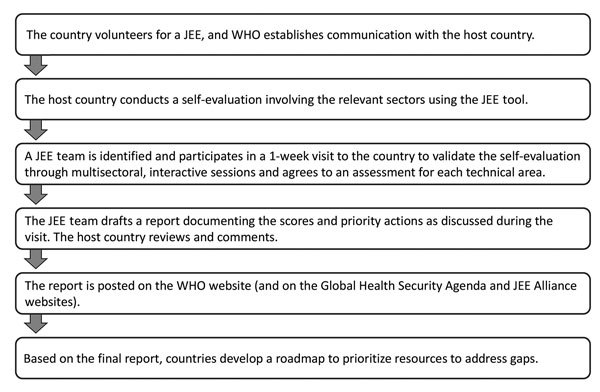
JEE process. Each JEE follows a standardized process that aligns with the principles of transparency, multisectoral engagement, and public reporting of the International Health Regulations 2005 (*2*) and the Global Health Security Agenda (https://www.ghsagenda.org/). JEE, Joint External Evaluation; WHO, World Health Organization.

**Figure 2:**
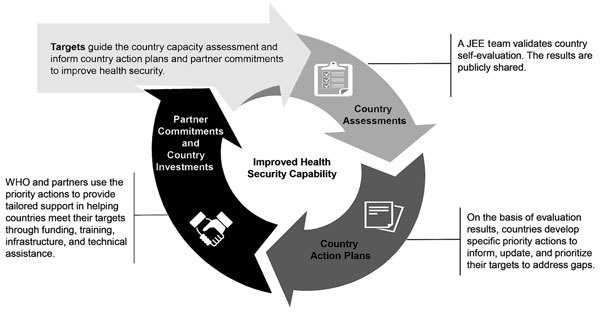
JEE continuum iterative process to identify and fill gaps in addressing requirements for each indicator under 19 technical areas. Each JEE follows a standardized process that aligns with the principles of transparency, multisectoral engagement, and public reporting of the International Health Regulations 2005 (*2*) and the Global Health Security Agenda (https://www.ghsagenda.org/). The process to improve health security capacity requires continuous evaluation of capabilities and (re)alignment of resources. JEE, Joint External Evaluation; WHO, World Health Organization.

Countries volunteer for JEEs by submitting a written request to WHO through their WHO representative or through the regional IHR coordinator at their WHO regional office. The JEE process is part of a continuum to strengthen countries’ ability to prevent, detect, and respond to health emergencies (Figures 1,2), which includes a self-assessment and external evaluation, simulation exercises, after-action reviews when an actual event occurs, and development of a national action plan and implementation. The requesting countries use the JEE tool to conduct a self-assessment involving all relevant sectors (including food and agriculture, animal health, and security sector). The countries then share the findings with the JEE Secretariat or with the WHO regional office, which assemble an external assessment team of international experts led by WHO and non-WHO experts. The results of the JEE self-assessment are shared with the external assessment team in advance of their week-long independent assessment. The external assessment team comprises ≈8–12 internationally recognized experts from multiple sectors. The mission typically lasts 1 week and comprises an internal briefing; meetings and consultations; field visits; and a final briefing to the primary JEE sector-relevant ministries, partners, civil society, and others. The external assessment team reviews the JEE self-assessment with the host country through 19 facilitated, multisectoral discussions between host country experts and the external assessment team. The JEE process brings together a multisectoral approach (e.g., animal and human health, food and agriculture, and security and law enforcement), enabling engagement and cooperation, often for the first time, of these disparate but health-related country experts and policy makers. Strengths, vulnerabilities, scores, and 3–5 priority actions for each of the 19 technical areas are jointly developed based on the standards in the JEE tool.

At the completion of the assessment, the JEE team presents its findings, along with recommended priority actions and capacity score, to the leaders of the line ministries and policy makers in the country. A final report is developed, shared with the country, and posted publicly. The country is expected to use the JEE report and other relevant assessments to develop a national action plan for health security or update an existing national action plan with associated costs so that compliance gaps can be addressed through domestic resources in collaboration with donors, partners, multilateral agencies (e.g., GHSA partnering countries, WHO, OIE, and FAO), and the public–private sector through technical assistance, funding support, or both (*11*).

## Completed JEEs

**Figure 3:**
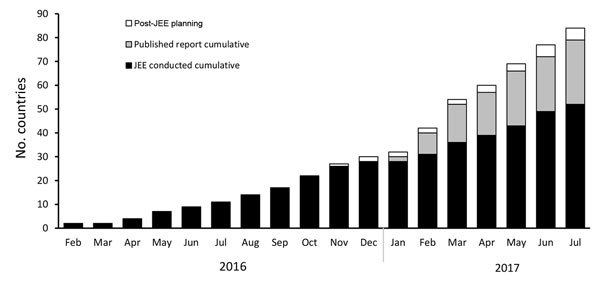
JEE scale-up over time. Each JEE follows a standardized process that aligns with the principles of transparency, multisectoral engagement, and public reporting of the International Health Regulations 2005 (*2*) and the Global Health Security Agenda (https://www.ghsagenda.org/). JEE, Joint External Evaluation.

By the close of the 70th World Health Assembly meeting (May 22–31, 2017) in Geneva, 41 countries had completed a JEE; 11 additional countries completed a JEE as of July 19, 2017. A total of 27 JEE reports (with the remaining under development) were posted on the WHO website (Figure 3) (https://extranet.who.int/spp/) as well as at the GHSA website (*12*). As of July 19, 2017, a total of 52 countries had completed a JEE, and an additional 25 countries are scheduled to complete a JEE by the end of 2017. In addition, the 6 GHSA countries that had previously completed an external GHSA pilot assessment of their capacities across the 11 GHSA action packages have now developed plans to complement their GHSA evaluation with a full JEE to complete the external assessment across all 19 IHR 2005 core capacities.

## Use of JEE Findings

The Strategic Partnership Portal, developed and hosted by WHO, is a member state–mandated information-sharing Web portal designed to enhance communication between countries, donors, partners, and WHO to better inform financial and technical support provided to countries. It is intended to monitor and map all contributions (e.g., financial, technical, in-kind, and in-service) from donors and partners to facilitate alignment of in-country efforts to address gaps and priorities and to reveal possibilities for future collaboration. The Strategic Partnership Portal is a 1-stop portal to facilitate sharing of information about current and future activities and investments to enable a more coherent, transparent, coordinated approach and more informed resource allocation decisions (*12*).

## Standardization and Quality Assurance of JEEs

In July 2016, WHO convened a JEE working group comprising members from WHO, CDC, the US Department of Agriculture, and the Government of Finland. The group examined lessons learned and best practices from the first 10 JEEs to ensure the standardization of the JEE implementation process and maintain high-quality evaluations and results, while rapidly scaling up JEE missions to meet countries’ demands.

### Rostering SMEs

The Government of Finland, with the WHO Secretariat, led development of a consolidated global roster of SMEs, working with all 6 WHO regional offices, the Global Outbreak Alert and Response Network Secretariat, and the IHR rosters of experts, as well as the GHS country steering committee, to identify appropriate and highly qualified SMEs to support the JEE missions. The Government of Finland, the Government of Germany, CDC, FAO, and OIE provided substantial technical support through their technical experts, as well as financial support for travel and logistics. JEE mission team leads were selected primarily from Finland, CDC, US Department of Agriculture, OIE, FAO, and WHO and initially comprised technical staff who were engaged in the JEE tool development. Currently, the consolidated JEE list of experts comprises ≈400 technical experts from government agencies, multilateral organizations, and academic institutions worldwide (https://extranet.who.int/spp/list-of-experts) (*12*).

### Staffing JEE Country Teams

The JEE working group developed principles for composing the independent experts’ country teams to ensure that teams have appropriate professional experience; gender and geographic representation balance; organizational diversity; and a mixture of new and experienced JEE participants for a transparent, objective, and credible outcome. WHO developed standard operating procedures for rostering JEE country teams to ensure standardized methods to guide the formation of the external country teams’ composition.

### Team Lead Training

After the initial JEEs were conducted and the working group reviewed lessons learned at the July 2016 meeting, it became evident that a strong team lead was an important contributor to a successful mission with a timely and accurate report with 3–5 specific priority actions for each of the 19 technical areas. The JEE working group developed a participatory team lead training, piloted at the WHO regional office in Brazzaville, Congo, during October 18–20, 2016; a total of 17 team leads from various key partners were trained. WHO reviewed and refined the training design and materials, and the second team lead training occurred in Lyon, France, during January 31–February 1, 2017; an additional 23 team leads were trained. Team leads who have been trained in facilitation, a common approach to applying the JEE tool, and development of final scoring enhances the standardization of results across country missions and comparability over time.

### External Evaluation Team Member Orientation

Another component of strengthening and standardizing the JEE missions and results is to ensure the external evaluation team members have a common understanding of the JEE process, mission requirements, and familiarity with the tool itself. With assistance from CDC and working group partners, WHO developed an online orientation to better prepare external evaluation team members (i.e., SMEs), enabling them to review the self-guided materials before participating on a country mission. The JEE team online orientation is available for rostered SMEs on the WHO online learning site (https://extranet.who.int/hslp/training/enrol/index.php?id=116).

### Country Self-Assessment Support

A strong and thorough country self-assessment is critical to obtaining high-quality JEE results. The external team reviews and validates the country’s self-assessment using the JEE tool and an incomplete, superficial, or less-than-timely country self-assessment can make it difficult for the external evaluation team to accurately assess, or appropriately recognize, a country’s health systems’ capacities if documentation and confirming evidence is lacking. The WHO EMRO piloted a JEE orientation workshop to support participating countries that provided training in the JEE tool and process, an approach that has proven highly successful (*13*). A key factor in maintaining high-quality evaluations was developing country orientation materials and providing on-site support for the self-assessment process by conducting an orientation workshop in each country. The WHO Secretariat developed guidance and materials based on the EMRO model to provide support and assistance to countries in implementing their self-assessment, an essential element in high-quality, timely self-assessments.

### JEE Tool Interpretation Guidance

As the number of JEEs grew and an increasing number of team leads and SMEs used the tool in real-world settings, ensuring that teams interpreted the tool consistently became important. In addition to the in-person team lead training and online SME orientation, WHO, with input from the JEE working group, developed a separate guidance tool to clarify areas and language that caused confusion or commonly elicited questions. Feedback on the tool and its use was solicited from JEE team members, technical experts, and the regional offices engaged in implementing the JEE. Most feedback received was incorporated into the Tool Interpretation Guide, scoring recommendations, guiding discussion questions, and expanding the glossary to ensure consistency in tool use and to maintain the tool’s integrity and the validity of already conducted JEEs and the ability to measure future progress in these countries. However, certain elements of the tool might need to be modified to resolve outstanding concerns discussed during the April 19–21, 2017, WHO consultation on the JEE tool. For example, 2 indicators on finance (1 on routine financing and 1 on emergency financing) will be added to the JEE’s National Legislation, Policy, and Finance technical area. In addition, referencing and linking with other technical areas will be improved, including scoring issues related with human and animal health technical areas.

## Discussion

The completion of 52 JEEs and the planning of 25 additional JEEs provide evidence for growing support and interest by WHO member states to volunteer for the JEEs. However, there are also technical limitations related to the JEE tool and the need for advocacy and communication of the JEE process to countries. WHO has obtained systematic feedback from participants on JEE teams related to the tool’s technical limitations and challenges associated with interpretation and scoring related to overlapping technical areas. WHO convened a consultation during April 19–21, 2017, involving relevant multisectoral partners and agency representatives, including member state partners who have undergone JEEs, to obtain recommendations to address these limitations and challenges.

WHO headquarters and regional offices and organizations, such as CDC, have developed communication materials that can be shared with countries potentially interested in volunteering for JEEs to provide them with information about advantages associated with JEEs and transparency of the reports. This transparent and collaborative approach has helped with strengthening existing collaborations and in establishing possible new collaborations and technical partnerships with potential public and private partners and donors willing to provide technical or financial assistance to address the gaps identified through JEEs. This well-coordinated implementation style also has been instrumental in establishing a “twinning process” between 2 countries, whereby 1 country establishes a technical partnership with another to provide assistance.

Sustaining the momentum for conducting and periodically repeating JEEs is critical to the success of the IHRMEF. To ensure coordination, management, and sustainability for the JEE process, WHO has established the JEE Secretariat at WHO headquarters in Geneva. Some member states and private partners and donors have provided the funding resources. Also, in support of the work of the WHO JEE Secretariat and ensuring the process continues, GHSA has created entities to support and accelerate the IHR 2005 implementation. These entities include the Alliance for Country Assessments for Global Health Security and IHR Implementation and an Alliance Advisory Group. The Alliance for Country Assessments for Global Health Security and IHR Implementation (https://www.jeealliance.org/) is an open partnership platform for facilitating multisectoral collaboration on health security capacity building and IHR implementation. The Alliance Advisory Group is drawn from Alliance members: 12 countries (2 from each WHO region); 4 nongovernment organizations and foundations; and 4 multilateral organizations. The Alliance Advisory Group members for the first 2-year term are the countries of Australia, Bangladesh, Cambodia, Finland, Georgia, Indonesia, Pakistan, Peru, Saudi Arabia, Senegal, Uganda, and the United States; the Bill and Melinda Gates Foundation; the Elisabeth R. Griffin Foundation (Chair of the GHSA NGO Consortium); the No More Epidemics Campaign; and the Training Programs in Epidemiology and Public Health Interventions Network. WHO, OIE, FAO, and the World Bank are permanent members.

Global health security relies on all countries working together in the spirit of transparency and mutual accountability to develop and maintain the core capacities required under the IHR 2005 implementation. Achieving implementation entails all member states having the capacity to prevent and to rapidly detect, verify, notify, and respond effectively to all public health threats while limiting the international spread of disease and its effect on travel and trade. Measuring progress toward implementation is therefore critical for efforts aimed at enhancing global health security. Completing a JEE demonstrates a country’s commitment to developing capacities required under the IHR 2005 and supports countries in establishing an objective baseline assessment of their public health capacities; identifies strengths and limitations within their health systems; and enables prioritizing opportunities for capacity development in disease prevention, detection, and response across all sectors, including all 19 technical areas and all hazards. The comprehensive, all-hazard assessment of capacities can inform a country’s roadmap/action plan, guide allocation of national resources, and engage current and prospective donors and partners to effectively target resources and technical assistance. Although JEEs provide an objective and transparent assessment, the effect of the JEEs depends on rapid development of a country-owned and country-led post-JEE national action plan for health security and its implementation to address the gaps by the country itself, as well as through support from donors and public and private partners. Member states are encouraged to conduct annual self-assessments using the JEE tool and are expected to conduct the JEE once every 4–5 years. Countries are encouraged to use the other voluntary components of IHRMEF (i.e., after-action review and simulation exercises) to provide qualitative assessment of IHR functionality and performance to validate plans, develop and practice staff competencies, and ascertain whether gaps identified during an actual public health event or tabletop exercise are addressed. Collectively, the IHRMEF can help measure progress and realign country plans as needed and report progress on implementation as part of annual reporting (*14*,*15*). Countries and partners need to commit to working together to implement national plans of action expediently and effectively to ensure impactful progress in addressing gaps and deficiencies. The JEE is a valuable mechanism to facilitate and measure progress toward IHR 2005 implementation and thereby enhance global health security.
